# State of the Art in Combination Immuno/Radiotherapy for Brain Metastases: Systematic Review and Meta-Analysis

**DOI:** 10.3390/curroncol29050244

**Published:** 2022-04-22

**Authors:** Masoumeh Najafi, Amin Jahanbakhshi, Marzieh Gomar, Cinzia Iotti, Lucia Giaccherini, Omid Rezaie, Francesco Cavallieri, Letizia Deantonio, Lilia Bardoscia, Andrea Botti, Angela Sardaro, Salvatore Cozzi, Patrizia Ciammella

**Affiliations:** 1 Skull Base Research Center, Iran University of Medical Sciences, Tehran 1997667665, Iran; najafi.mas@iums.ac.ir; 2 Stem Cell and Regenerative Medicine Research Center, Iran University of Medical Sciences, Tehran 1997667665, Iran; jahanbakhshi.a@iums.ac.ir; 3 Radiation Oncology Research Center, Iran Cancer Institute, Tehran University of Medical Sciences, Tehran 1416753955, Iran; m.gomar1365@gmail.com; 4 Radiation Therapy Unit, Azienda USL-IRCCS di Reggio Emilia, 42123 Reggio Emilia, Italy; cinzia.iotti@ausl.re.it (C.I.); lucia.giaccherini@ausl.re.it (L.G.); patrizia.ciammella@ausl.re.it (P.C.); 5 Hematology-Oncology Department, Jam Hospital, Tehran 1997667665, Iran; omid55r@yahoo.com; 6 Neurology Unit, Neuromotor & Rehabilitation Department, Azienda USL-IRCCS di Reggio Emilia, 42123 Reggio Emilia, Italy; francesco.cavallieri@ausl.re.it; 7 Radiation Oncology Clinic, Oncology Institute of Southern Switzerland (IOSI), 6500 Bellinzona, Switzerland; letizia.deantonio@eoc.ch; 8 Radiation Oncology Unit, S. Luca Hospital, Healthcare Company Tuscany Nord Ovest, 55100 Lucca, Italy; liliabardoscia@gmail.com; 9 Medical Physics Unit, Azienda USL-IRCCS di Reggio Emilia, 42123 Reggio Emilia, Italy; andrea.botti@ausl.re.it; 10 Section of Radiology and Radiation Oncology, Interdisciplinary Department of Medicine, University of Bari “Aldo Moro”, 70124 Bari, Italy; angela.sardaro@uniba.it

**Keywords:** brain metastases, immunotherapy, radiotherapy, melanoma, non-small-cell lung carcinoma

## Abstract

Objectives: Common origins for brain metastases (BMs) are melanoma, lung, breast, and renal cell cancers. BMs account for a large share of morbidity and mortality caused by these cancers. The advent of new immunotherapeutic treatments has made a revolution in the treatment of cancer patients and particularly, as a new concept, if it is combined with radiotherapy, may lead to considerably longer survival. This systematic review and meta-analysis aimed to evaluate the survival rate and toxicities of such a combination in brain metastases. Methods: To perform a systematic review of the literature until January 2021 using electronic databases such as PubMed, Cochrane Library, and Embase; the Newcastle–Ottawa Scale was used to evaluate the quality of cohort studies. For data extraction, two reviewers extracted the data blindly and independently. Hazard ratio with 95% confidence interval (CI), fixed-effect model, and inverse-variance method was calculated. The meta-analysis has been evaluated with the statistical software Stata/MP v.16 (The fastest version of Stata). Results: In the first step, 494 studies were selected to review the abstracts, in the second step, the full texts of 86 studies were reviewed. Finally, 28 studies were selected consisting of 1465 patients. The addition of IT to RT in the treatment of brain metastasis from melanoma and non-small-cell lung carcinoma was associated with a 39% reduction in mortality rate and has prolonged overall survival, with an acceptable toxicity profile. The addition of IT to RT compared with RT alone has a hazard ratio of 0.39(95% CI 0.34–0.44). Conclusions: A combination of immuno/radiotherapy (IR) for the treatment of patients with BMs from melanoma and non-small-cell lung carcinoma has prolonged overall survival and reduced mortality rate, with acceptable toxicity. In terms of timing, RT seems to have the best effect on the result when performed before or simultaneously with immunotherapy.

## 1. Introduction

Development of brain metastases (BMs), often associated with extracranial progression of the disease, determines a poor prognosis with a few months of overall survival rate [1]. The most common causes of BMs are breast cancer, lung cancer, melanoma, and renal cell carcinoma (RCC) [2]. Compared with these cancers, melanoma has a high propensity to metastasize to the brain and is associated with the highest BMs ratio [3]. Historically, treatment options for BMs were limited and were generally used for palliative purposes. When the concept of local treatment for the oligometastatic disease was introduced, the therapeutic approach changed considerably. Local treatment, consisting of surgery and radiotherapy, became the standard of care and the limitation of systemic treatments delivery through the blood–brain barrier was overcome [4,5,6,7]. Two types of radiotherapy are commonly used for BMs treatment, including whole-brain radiotherapy (WBRT) and stereotactic radiotherapy (SRT) [8].

During the last ten years, another revolutionary change was made in this setting, namely the advent of new systemic treatments, especially modern immunotherapy (IT) aimed at immune checkpoint pathways (PD1/PD-L1 and CTLA-4). Immunotherapy has reduced the progression of primary melanoma, NSCLC, and RCC [9,10,11]. Studies have shown that IT can achieve long-term survival (20–30%) and long-term benefits [12,13]. Few studies have been conducted on the effectiveness of IT against BMs. Goldberg et al. reported promising results in 52 untreated BMs patients with melanoma or NSCLC. There were only a few patients who failed to respond to pembrolizumab [14]. Long et al. showed that in 63 patients with previously untreated BMs, a high proportion of patients achieved an intracranial response with the combination of nivolumab and ipilimumab (20–46%) [15].

Consequently, the combination of RT and IT became attractive for researchers. Local radiotherapy can theoretically enhance response to immunotherapy by cell lysis and release of cancer antigens, increasing their presentation to immune cells, production of inflammatory cytokines, inhibiting immune-suppressing cells, and activating a specific anti-tumor response [16,17,18,19]. RT may alter the function of BBB and allow IT drugs and immune cells to penetrate the BBB [20]. The optimal time for administration of RT varies between different studies. An advantage has been reported for implementation before IT to six months after IT. Thus, the impact of timing remains unclear.

To date, a comprehensive conclusion on the optimal combination of IT and RT for brain metastases in terms of dose and timing has not been achieved. Existing studies have shown that the combination of IT and RT for the treatment of BMs can be considered a safe and promising strategy [21,22,23,24]. The objective of the present systematic review and meta-analysis study is to evaluate the clinical outcomes, i.e., overall survival (OS) and toxicity, of a combination of immuno/radiotherapy (IR) for brain metastases.

## 2. Methods

### 2.1. Search Strategy

MEDLINE, PubMed, Cochrane Library, Embase, ISI, and Google Scholar were used as electronic databases to perform a systematic literature review until January 2021. Therefore, a software program (Endnote X8) was used for managing the electronic titles. Searches were performed with following mesh terms:

(“Brain Neoplasms/blood”[Mesh] OR “Brain Neoplasms/chemistry”[Mesh] OR “Brain Neoplasms/classification”[Mesh] OR “Brain Neoplasms/complications”[Mesh] OR “Brain Neoplasms/epidemiology”[Mesh] OR “Brain Neoplasms/etiology”[Mesh] OR “Brain Neoplasms/history”[Mesh] OR “Brain Neoplasms/immunology”[Mesh] OR “Brain Neoplasms/mortality”[Mesh] OR “Brain Neoplasms/radiotherapy”[Mesh] OR “Brain Neoplasms/surgery”[Mesh] OR “Brain Neoplasms/therapy”[Mesh])) OR “Cerebral Ventricle Neoplasms”[Mesh]) OR “Choroid Plexus Neoplasms”[Mesh]) OR “Infratentorial Neoplasms”[Mesh]) OR “Brain Stem Neoplasms”[Mesh]) OR “Cerebellar Neoplasms”[Mesh]) OR “Brain Neoplasms”[Majr:NoExp]) OR “Brain Neoplasms”[Mesh]) OR (“Neoplasm Metastasis”[Mesh] OR “Lymphatic Metastasis”[Mesh])) AND (“Radiosurgery/adverse effects”[Mesh] OR “Radiosurgery/methods”[Mesh] OR “Radiosurgery/therapy”[Mesh])) OR “Radiotherapy”[Mesh]) OR (“radiotherapy” [Subheading] OR “Radiosurgery”[Mesh])) AND (“Immunotherapy/adverse effects”[Mesh] OR “Immunotherapy/complications”[Mesh] OR “Immunotherapy/immunology”[Mesh] OR “Immunotherapy/methods”[Mesh] OR “Immunotherapy/therapy”[Mesh])) OR “Immune Checkpoint Inhibitors”[Mesh]) OR (“Immunotherapy”[Mesh] OR “Immunotherapy, Active”[Mesh])) OR “CTLA-4 Antigen”[Mesh].

This systematic review has been conducted based on the critical consideration of the PRISMA [25].

### 2.2. Selection Criteria

#### 2.2.1. Inclusion Criteria

1. Randomized controlled trials, controlled clinical trials, and prospective and retrospective cohort studies. 2. Efficacy and safety of RT. 3. WBRT or SRT. 4. Adult patients. 5. Patients with BM from solid tumors. 6. Immunotherapy. 7. No language restrictions. 8. Brain metastasis.

#### 2.2.2. Exclusion Criteria

1. In vitro studies, reviews, case-control studies, case reports, and animal studies. 2. Incomplete or inconsistent data.

### 2.3. Data Extraction and Method of Analysis

The extracted data consisted of years, study design, sample size, primary tumor, N° BMs, and overall response rate. The Newcastle–Ottawa Scale (NOS) [26,27] was used to assess the quality of all eligible studies. This scale measures three dimensions (selection, comparability of cohorts, and outcome) with a total of 9 items. In the analysis, any studies with NOS scores of 1–3, 4–6 and 7–9 were defined as low, medium, and high quality, respectively. Any disagreement between reviewers was resolved by discussion with the whole study team. 

For data extraction, two reviewers extracted data from the abstract and full text of the studies included, blindly and independently. Before the screening, kappa statistics were carried out in order to verify the agreement level between the reviewers. The kappa values were higher than 0.80.

A hazard ratio with 95% confidence interval (CI), fixed effect model, and Mantel–Haenszel method were calculated. Random effects were used to deal with potential heterogeneity, and I^2^ showed heterogeneity. I^2^ values above 50% signified moderate-to-high heterogeneity. The meta-analysis was evaluated with the statistical software Stata/MP v.16 (the fastest version of Stata). By the time of completion of the work, registration in PROSPERO was not a routine local research protocol. So, we do not have a registration number, although web search was conducted to avoid repetition.

## 3. Results

**Figure 1:**
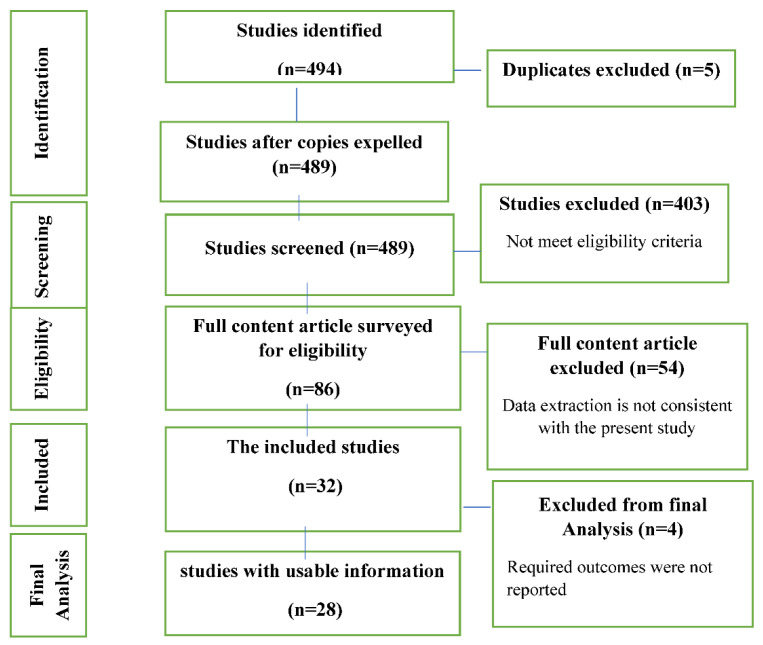
Study Attrition.

According to the purpose of the study, in the initial search with keywords, 494 articles were found. In the first step, 489 studies were selected to review the abstracts. Then, studies that did not meet the inclusion criteria were excluded from the analysis. In the second step, the full text of 86 studies was reviewed. Finally, 28 studies were selected (Figure 1) with a total number of 1465 patients (Table 1).

### 3.1. Characteristics

The total number of patients was 1465, consisting of melanoma (*n* = 1273), non-small-cell lung cancer (*n* = 180), and renal cell carcinoma (*n* = 12). The overall response rate of BMs metastases ranged from 4–76% in ten studies. (Table 1). Radiotherapy modalities included SRT or stereotactic radiosurgery (SRS) in 19 studies, WBRT in one study, and both SRT and WBRT in 12 studies. The mean dose range used for SRT and WBRT were between 20–25 Gy and 30 Gy, respectively. The timing between RT and IT and the number of cycles and drugs are described in Table 2. 

### 3.2. Bias Assessment

According to the NOS tool, eight studies had a total score of 5/9, eight studies had a total score of 6/6, four had a total score of 7/9, and twelve had a total score of 8/9. Sixteen studies had medium quality and sixteen studies had a low risk of bias (high quality) (Table 3).

## 4. Toxicity

Toxicity equal to or greater than G4 is reported in a few studies and represents a rather rare adverse event. However, mild to moderate toxicities (G1-3) were frequently described in 22 studies with 842 patients. Fatigue was reported in 11% of participants (*n* = 44) [51]. Dermatologic toxicity is the most common side effect secondary to immunotherapy (7–48%). Liniker et al. [48] reported 5% and 10% of Stevens–Johnson syndrome and cutaneous rash, respectively. In 5–41% of patients, cognitive changes predominantly in patients undergoing WBRT were reported. Bleeding was observed in 18–28% of patients. Radionecrosis was described in 15 of 33 studies (1–27.6%). Headache was observed in 4–26% of patients. Schapira et al. [31] reported ataxia in 4.2% and diarrhea, nausea, and anorexia were observed in 10–31%, 5–9% and 4–5% of patients, respectively. 

## 5. Overall Survival of Radiotherapy + Immunotherapy vs. Radiotherapy Alone 

**Figure 2:**
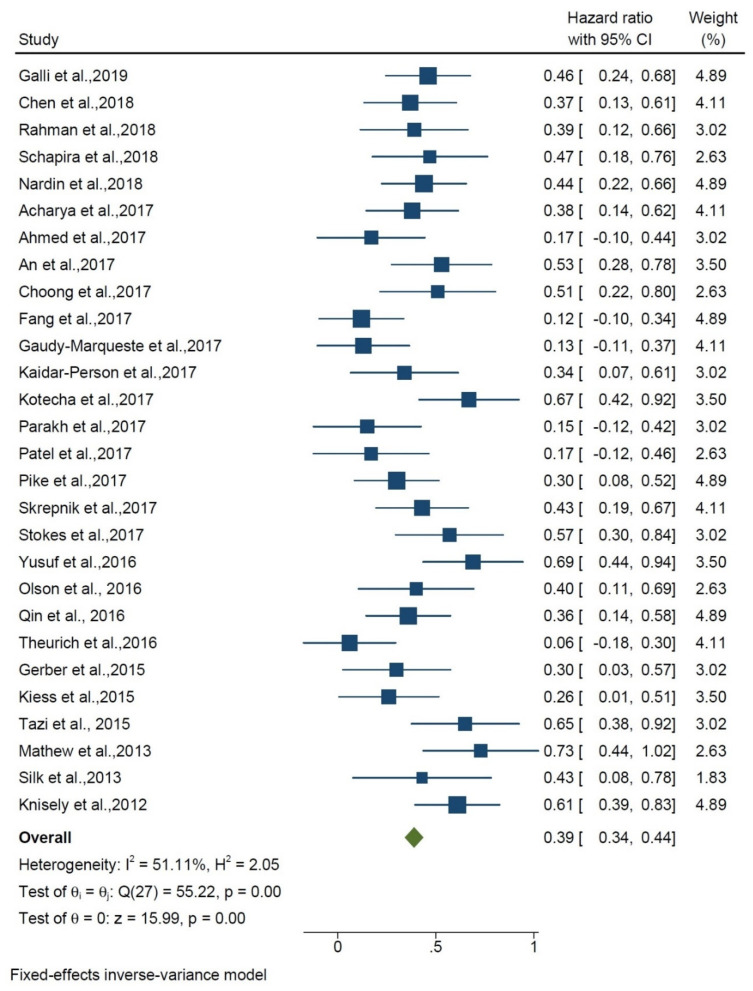
Forest plot shows overall survival of RT + IT vs. RT alone.

The hazard ratio for the effect of the addition of IT to RT compared with RT alone was 0.39 (HR, 0.39 95% CI 0.34, 0.44) among 28 studies, and heterogeneity was found (I^2^ = 51.11%; *p* < 0.01) (Figure 2). Stereotactic radiation therapy represents the treatment of choice in BMs, while the use of WBRT is reported in only five papers. The average dose varied a lot, from 18 to 30 Gy. RT techniques (SRS vs. WBRT) were chosen at the discretion of the physician, based on the number and size of metastases. Much attention was paid to the timing between RT and IT. Although there is no unanimous agreement, most of the authors consider 30 days between RT and IT to have a synergistic effect. Chen et al. reported that SRS–SRT with concurrent IT was associated with improved OS compared with SRS–SRT alone (PZ.002; hazard ratio (HR), 2.69) and compared with nonconcurrent treatment (PZ.006; HR, 2.40) on multivariate analysis. Concurrent therapy was defined as within two weeks before or after SRS/SRT. They demonstrated that concurrent SRS–SRT and IT may be associated with a reduced incidence of new intracranial metastases, as well as a favorable survival outcome.

## 6. Overall Survival of 1 and 2 Years

**Figure 3:**
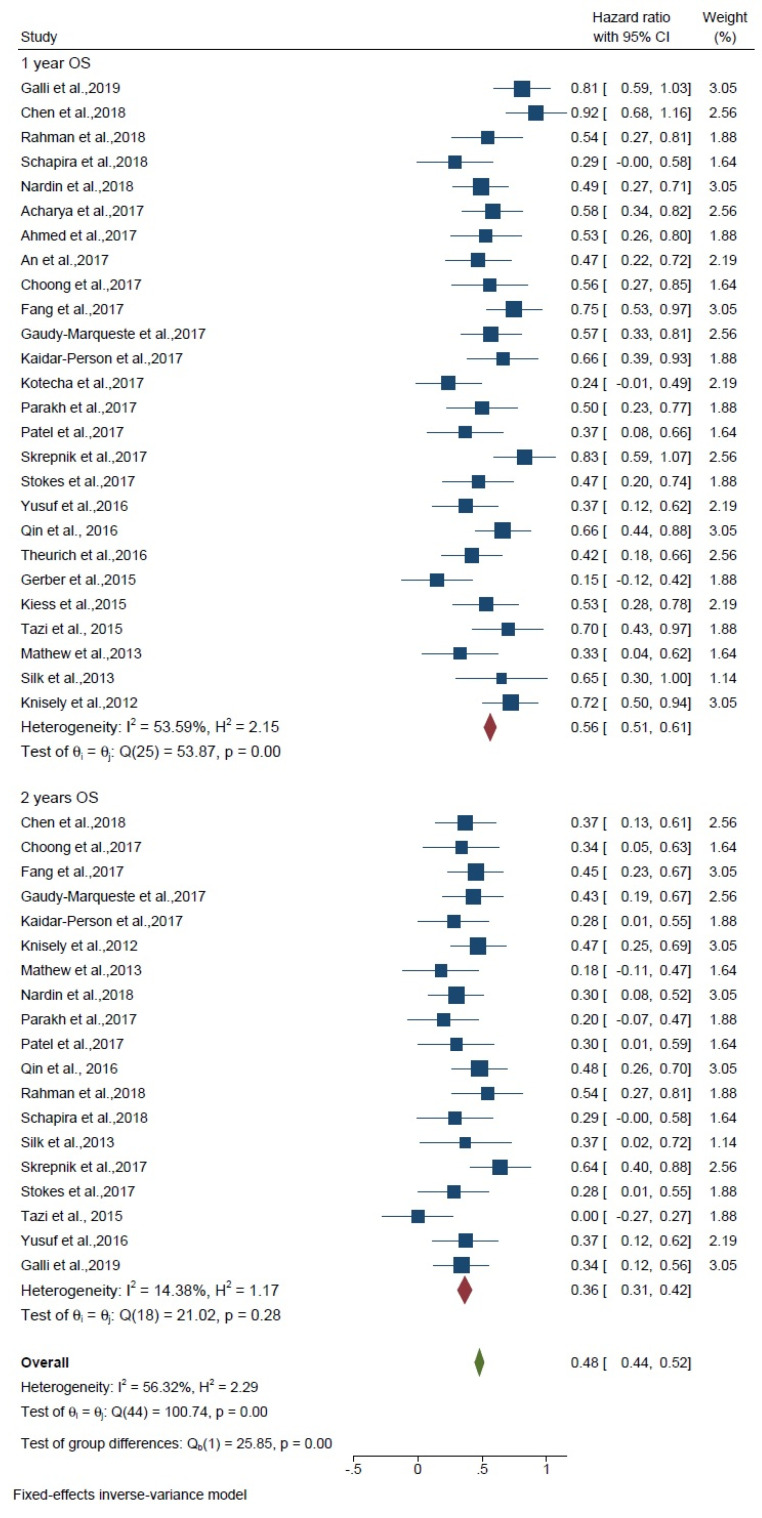
Forest plot shows overall survival of 1 and 2 years.

Overall survival was assessed separately for 1 and 2 years, and these are compared with each other. For 1-year overall survival, the overall HR was 0.56 (95% CI: 0.51–0.61) among 16 studies, and heterogeneity was found (I^2^ = 53.59%; *p* < 0.01). For 2-year OS, the overall HR was 0.36 (95% CI: 0.31–0.42) among 19 studies and heterogeneity was found (I^2^ = 14.38%; *p* = 0.28). The hazard ratio of the subgroup meta-analysis between 1 and 2 years was 0.48 (HR, 0.23 95% CI 0.44, 0.52). The test of difference group was *p* < 0.01; no significant difference was observed between the groups (Figure 3). Therefore, the effect of treatment was not different for 1- or 2-year overall survival.

## 7. Discussion

Brain metastasis usually carries poor prognostic foresight. Sperduto et al. [59] showed that the worst and the best overall survival for metastatic melanoma are 5 and 34 months, respectively. Radiation therapy is a key component in the management of BM. The conventional management of BMs includes resection when feasible and WBRT, especially when multiple lesions are seen. Cell-survival curves generated after exposing metastatic cells to doses of ionizing radiation commonly used in WBRT have shown that these cells are often able to repair damage from small radiation doses. Therefore, SRS with a single higher dose of radiation appears to be better suited for addressing brain metastases [60]. SRS alone leads to high local control (70–80%), and OS ranged between 8 to 10 months, depending on the number and size of intracranial metastases [61].

RT induces damage to cancer DNA resulting in a cytotoxic effect, that is cell lysis leads to the release of cancer antigens, increasing their presentation to immune cells and activating a specific antitumor response. On such a speculative basis, it was hypothesized that RT and IO could have a synergistic effect, with an increase in drug efficacy as a consequence of local RT. 

Not only is immunotherapy for brain metastasis a rapidly developing treatment modality, but there are also a lot of pieces of evidence that encourage physicians to use immunotherapy for BMs. A recent study by Téglási et al. [62] showed that there is a strong association between PD-L1 expression of primary non-small-cell lung cancer and their BMs, therefore, PD-L1 positivity in the primary tumor could serve as a therapeutic criterion even for brain metastases. Takamori et al. [63] found that 21.9% of patients with NSCLC showed PD-L1 positivity in BMs and reported that the PD-L1 expression in BMs may be associated with local recurrence following surgery, underlining the possible determined role of IT. Several studies reported the results of immunotherapy for BM either as monotherapy or combination therapy. Wolchok et al. [64] reported 3-year overall survival outcomes with combined nivolumab and ipilimumab in advanced melanoma. The overall survival rate at 3 years was 58% in the nivolumab-plus-ipilimumab group and 52% in the nivolumab group, as compared with 34% in the ipilimumab group. 

A valuable strategy for BMs with melanoma and NSCLC is to combine IT with RT. Strong preclinical and clinical justifications have been reported for combining treatment, however, the clinical application of this combination is not well established, mainly due to the lack of high-quality data from prospective studies and the presence of very heterogeneous studies on the efficacy of immunotherapy in brain lesions [54,65]. There is, also, insufficient information about the safety of combining RT with IT, especially in elderly patients, because data are limited [66]. In the present study, we performed a systematic review and meta-analysis to evaluate the clinical outcomes of combining immunotherapy and radiotherapy for brain metastases and assessed the toxicities associated with this treatment. 

The results of the meta-analysis show that the hazard ratio of effect of addition of IT to RT compared with RT alone was 0.39. Median overall survival of about 16 months from initiation of any treatment was observed and 1- and 2-year survival rates were strictly correlated (56% and 36%, respectively). The overall results show that when adding IT to RT, the risk of death is reduced by about 50%. This benefit could be due to its effect on intracranial and/or systemic disease, which cannot be elucidated by this study and needs further investigation. Goldberg et al. [15] showed that the average overall survival is about 16 months, which is consistent with the results of the present study. The quality of the studies was moderate to high. High heterogeneity was observed between the studies, so the prognosis of BMs is widely heterogeneous and depends on several factors. Goldberg et al. [15] showed that patients with lung or melanoma BMs had an overall intracranial disease response of 18% and 33%, respectively. 

There are clinical experiences presenting contradictory data, with some retrospective case series documenting a synergy between RT and IT and others showing no benefit from combined treatments. For example, Silk et al. [30] reported a significant OS prolongation in patients treated with ipilimumab, in comparison with control cases receiving RT alone (19.9 vs. 4.0 months, *p* = 0.009). On the other hand, Patel et al. [12] documented no differences in PFS and OS in a similar case series. Recent data about anti-PD1 agents in concomitance with RT are more encouraging. For example, Choong et al. [23] reported a promising OS of 20.4 months with SRT administered within 6 weeks from an anti-PD1. Gaudy-Marquette et al. [14] found a particularly strong synergy with the combination of anti-PD1 and RT (median OS 14.8 months). Chen et al. [2] demonstrated that concurrent IT predicted for reduced probability of subsequent development of three new BMs in patients following SRS–SRT (PZ.045; odds ratio, 0.337).

Some authors speculated that the frequent administration of steroids to patients with intracranial disease, forced by the high prevalence of neurologic symptoms, might have weakened RT/IT immune response, thus hiding a potential synergistic effect. 

Toxicities: Many studies did not report toxicities, dose-volume information, comorbidities, and measures on quality of life. Kiess et al. administered concurrent SRS and ipilimumab for 15 patients and reported one G4 cardiopulmonary toxicity, one rash/pruritus G3, one hepatitis G3, two G3 CNS hematoma, and two seizures. Relative to nonconcurrent administration, simultaneous consumption of checkpoint inhibitors with SRS was also associated with an increment in the size of hematoma in irradiated lesions but showed that concurrent administration of SRS and immunotherapy leads to better outcomes in terms of response and survival [54]. Nardin et al. investigated retrospectively all trials on melanoma BMs treated with pembrolizumab plus SRS between 2012 and 2015. They found radiation necrosis in 6.8% of patients, unaffected by timing between SRS and pembrolizumab [33]. Yusuf et al. tried the combination of immunotherapy (ipilimumab or pembrolizumab) and SRS in 12 patients with melanoma BMs, and after 5 months, radionecrosis was observed in 16.7% of patients [48]. In another study by Chen et al. [28], 28 patients with brain metastases were treated concurrently with checkpoint inhibitors and radiosurgery (median dose 20 Gy in a single fraction). They reported 3% of G3 CNS toxicity and 1% of G3 immune-related toxicity, and this was not significantly different from conventional methods. Schapira et al. [31] reported ataxia (4.2%), moreover, surprisingly, there was an increase in G3 toxicity in patients who performed IT before RT, while they did not record an increase in toxicity in concurrent treatment.

Some studies declare that concurrent and nonconcurrent treatment with radiation and checkpoint inhibitors achieve better outcomes with no increased toxicity [28,29,31,32,46,48,54,67,68]. However, others warn of possible immune-related adverse events and a synergistic effect of radiotherapy and immunotherapy on toxicities [69]. According to our analysis, G1-3 toxicity was reported in about half of the patients and radionecrosis in fewer than 30% of patients. Radionecrosis, a major concern in brain radiotherapy and a source of significant morbidity and mortality in patients with brain metastases, is not a straightforward diagnosis. It can be confused with local recurrence and immune-related changes unless being studied histopathologically. Apart from this diagnostic challenge, other issues may have some role in different rates reported in the literature, often between 0 to 30%. Significantly higher rates of radionecrosis associated with immunotherapy in some studies may be the result of higher survival rates in this group, as radionecrosis is more prone to be seen after 6 months. Other studies suggested the contributing role of MAPK inhibitors’ administration before immunotherapy [41].

Similar studies: Trapani et al. published a systematic review on concurrent SRS or SRT and immunotherapy in which they indicated, again, an increase in local and regional control in concurrent therapy and no added toxicity. However, their study has a small number of patients (*n* = 252) related to 16 papers up to December 2018 [70]. Lehrer et al. published a meta-analysis in 2019 analyzing 534 patients from 17 studies. They reported higher OS, local and regional control in concurrent treatment with SRS and checkpoint inhibitors versus nonconcurrent treatment, but they could not analyze radionecrosis due to limited data [71]. Another meta-analysis by Petrelli et al. showed that the addition of IT to RT improves overall survival. RT given before IT may give superior results than reverse sequencing; in fact, RT given before IT may improve BBB permeability, thus allowing IT drugs to then penetrate the brain. They have analyzed 754 patients from 13 studies. They reported a hazard ratio of 0.54 with a 95% CI of 0.44 to 0.67 [72]. Rulli et al., in another meta-analysis of 15 trials, reported a longer PFS and OS in combined immunotherapy compared with mono-immunotherapy or targeted therapy [73]. In comparison, our study analyzed 28 studies consisting of 1465 patients, which came from a literature search up to January 2021. We reached a lower hazard ratio compared with Petrelli et al. but with a smaller range of confidence interval (HR: 0.39, 95% CI: 0.34–0.44).

Limitations and strengths: The included studies mainly consisted of patients with BMs from melanoma (1273/1465). So, the results may not be generalizable to patients with other primary tumors. Furthermore, the unknown mutation status of the melanomas and NSCLC tumors may be the source of bias. Moreover, there is considerable heterogeneity between study results, the sample size was small, uniformity in the study method was poor, and patient characteristics were different in terms of DS-GPA (diagnosis-specific graded prognostic assessment) score and tumor cells’ biology. In terms of toxicities, also, there is a lack of data in many studies, especially those considering long-term toxicities such as neurocognitive impairment. All studies were retrospective, which makes our work prone to biases. A search on clinicaltrials.gov shows that there are two prospective cohorts and seven randomized studies recruiting metastatic patients for IT and RT. These studies, hopefully, will provide high-quality evidence for a better conclusion.

Regarding the timing of IT and RT, there are variations in the definition of concurrent therapy. In the nonconcurrent therapy group, patients who receive IT after RT tend to have systemic progression, and those who receive RT after IT tend to have intracranial progression [28]. So, the sequence of IT and RT and background conditions indicated either or both therapies are potential sources of bias.

Altogether, this is the most up-to-date meta-analysis performed on studies up to early 2021; the overall survival was examined as a subgroup meta-analysis in terms of 1 year and 2 years; the quality of studies was medium and high, and the risk ratio was moderate to low. It was reported that the study with a high-risk ratio was not included in the study.

## 8. Conclusions

According to the results of the current meta-analysis, the addition of IT to RT is associated with a 39% reduction in mortality and prolonged overall survival, along with an acceptable toxicity profile. RT seems to have the best effect on the result when performed concurrently with immunotherapy. Therefore, at least in brain metastasis from melanoma and, to some extent, in non-small-cell lung carcinoma, the addition of immunotherapy to radiotherapy is a viable treatment option. It should be insisted here that the results of our study should be interpreted with caution due to the retrospective nature of studies and potential biases related to design, timing, and patients’ characteristics. Moreover, radiotherapy methods, doses, fractions, and combinations with new immunotherapy agents should be further designed in clinical trials. Moreover, the effect of sole immunotherapy, especially in melanoma, which is known to be radioresistant, can be compared with other treatment modalities in future studies.

## Figures and Tables

**Table 1:** Studies selected for systematic review and meta-analysis.

N	Study. Years	Study Design	Number of Patients	Mean Age	Primary Tumor	Average Number of Metastases (% of Patients)	Size	Overall Response Rate of Brain Metastases * (%)
1	Galli et al., 2019 [24]	R	36	59	Melanoma	8	NR	NR
2	Chen et al., 2018 [28]	R	79	59	NSCLC, Melanoma Kidney	2	NR	NR
3	Diao et al., 2018 [29]	R	59	61	Melanoma	NR	NR	76
4	Hubbeling et al., 2018 [30]	R	50	61	NSCLC	1: 60%; >3: 40%	MLD: 13 mm	NR
5	Rahman et al., 2018 [31]	R	35	66.7	Melanoma	2	MD: 9 mm	NR
6	Schapira et al., 2018 [32]	R	37	63	NSCLC	NR	MD: 6 mm	NR
7	Nardin et al., 2018 [33]	R	25	58	Melanoma	NR	MD: 16 mm	36
8	Acharya et al., 2017 [34]	R	18	61	Melanoma	NR	MV: 362 mm^3^	NR
9	Ahmed et al., 2017 [35]	R	17	60	NSCLC	NR	NR	NR
10	An et al., 2017 [36]	R	99	62	Melanoma	2	MV: 1.45 mm^3^	NR
11	Anderson et al., 2017 [37]	R	21	67	Melanoma	1.5	MD: 10 mm	32
12	Choong et al., 2017 [38]	R	39	64	Melanoma	2	NR	NR
13	Fang et al., 2017 [39]	R	137	57	Melanoma		MV: 122 mm^3^	NR
14	Gaudy-Marqueste et al., 2017 [40]	R	56	54.3	Melanoma	1: 30.3%; >3: 43.5%	NR	NR
15	Kaidar-Person et al., 2017 [41]	R	29	57	Melanoma	NR	MLD: 15 mm	NR
16	Kotecha et al., 2017 [42]	R	32	57	Melanoma	1: 34%; >3: 34%	MD: 9 mm	NR
17	Parakh et al., 2017 [43]	R	66	62	Melanoma	1: 10%; 2–4: 52%; >4: 38%	MSD: 23.5 mm	29
18	Patel et al., 2017 [44]	R	20	56.5	Melanoma	1–3: 90%; ≥3: 10%	NR	NR
19	Pike et al., 2017 [45]	R	85	63	NSCLC Melanoma RCC	NR	NR	NR
20	Skrepnik et al., 2017 [46]	R	25	68.5	Melanoma	NR	NR	4
21	Stokes et al., 2017 [47]	R	185		Melanoma	NR	NR	NR
22	Yusuf et al., 2016 [48]	R	18	63.8	Melanoma	NR	MD: 7.9 mm	NR
23	Liniker et al., 2016 [49]	R	27	63	Melanoma	7		33
24	Olson et al., 2016 [50]	R	26	63	Melanoma	NR	NR	NR
25	Qin et al., 2016 [51]	R	44	58	Melanoma	NR	NR	64.5
26	Theurich et al., 2016 [52]	R	46	62	Melanoma	NR	NR	NR
27	Gerber et al., 2015 [53]	R	13	64	Melanoma	NR	NR	11
28	Kiess et al., 2015 [54]	R	46	57	Melanoma	NR	MD: 8 mm	75
29	Tazi et al., 2015 [55]	R	10	65	Melanoma	NR	NR	NR
30	Mathew et al., 2013 [56]	R	25	62	Melanoma	>1: 84%; >4: 24%	MV: 0.6 mm^3^	NR
31	Silk et al., 2013 [57]	R	33	56	Melanoma	1–3	NR	27
32	Knisely et al., 2012 [58]	R	27	53	Melanoma	NR	NR	NR

R: retrospective study; MD: median diameter; MLD: median of the largest lesion’s diameter; MV: median volume; MSD: median sum of dimensions; NSCLC: non-small-cell lung carcinoma; RCC: renal cell carcinoma; NR: not reported. * most studies used response evaluation criteria in solid tumors (RECIST) as the indicator of response to treatment.

**Table 2:** Characteristics of local and systemic treatments.

N	Study. Years	Radiotherapy Type (%)	Dose (Gy)	Timing Radiotherapy (%)	Immunotherapy Drug	Number of Cycles	Main Toxicities (%)
1	Galli et al., 2019 [24]	SRS WBRT	SRS: 20–24 WBRT: 30	Concurrent: 100	Anti-CTLA4: 36 Anti-PD1: 4 BRAFi ± MEKi: 28	NR	NR
2	Chen et al., 2018 [28]	SRS (100)	20	Concurrent: 100	PI, NIVO or PEMBRO: NR	NR	Radionecrosis (3)
3	Diao et al., 2018 [29]	SRS (100)	20	Concurrent: 100	IPI: 100	4	Radionecrosis (2), hemorrhage (18)
4	Hubbeling et al., 2018 [30]	WBRT (58) or PBI (16) or SRS (70)	30	Before: 60	NIVO: 78; PEMBRO: 16; ATEZO: 8	9	Overall G3-4 (9) (SRS) & (10) (WBRT);
5	Rahman et al., 2018 [31]	SRS (100)	18	100: concurrent	IPI: 68; PEMBRO: 20; IPI + NIVO: 6; NIVO: 3; Other: 3	NR	Radionecrosis (14.3)
6	Schapira et al., 2018 [32]	SRS (100)	18		NIVO: 83.8	7	G3: ataxia (4.2), headache (4.2)
7	Nardin et al., 2018 [33]	SRS (100)	20	Before: 36 Concurrent: 38 After: 26	PEMBRO: 100	NR	G3 radionecrosis (12)
8	Acharya et al., 2017 [34]	SRS: 100	20	Concurrent: 6 After: 94	NIVO, PEMBRO or IPI: NR	4	Radionecrosis: 1
9	Ahmed et al., 2017 [35]	SRS: 82 SRT: 18	SRS: 20 SRT: 25	Before: 47 Concurrent: 27 After: 26	NIVO: 65 DURVALUMAB: 35	NR	NR
10	An et al., 2017 [36]	SRS: 100	20	After: 100	IPI: 100 PEMBRO: 100	NR	NR
11	Anderson et al.,2017 [37]	WBRT (14); SRS (52); Post surgery (33)	30	Concurrent: 100	PEMBRO: 100	4	NR
12	Choong et al., 2017 [38]	WBRT (38.9) SRS (73.1)	NR	Concurrent: 100	IPI: 72; anti-PD-1: 28	NR	Radionecrosis (2.8)
13	Fang et al., 2017 [39]	SRS or WBRT + SRS (100)	20	Before: 39	IPI: 87; PEMBRO: 9;	NR	Radionecrosis (27)
14	Gaudy-Marqueste et al., 2017 [40]	SRS (100)	NR	After: 61 Before: 47	IPI: 49; PEMBRO: 40	NR	NR
15	Kaidar-Person et al., 2017 [41]	SRS (100)	21	Before: 55	IPI: 65.5	NR	Radionecrosis (27.6); hemorrhage (24)
16	Kotecha et al., 2017 [42]	SRS (100)	NR	Before: 100	PD-1 or IPI: 100	NR	Radionecrosis (2)
17	Parakh et al., 2017 [43]	SRS (23) WBRT(30) Chir + RT (46)	NR	Before: 100	NIVO, PEMBRO: NR	NR	NR
18	Patel et al., 2017 [44]	SRS (100)	20	Before: 35 Concurrent: 5 After: 60	IPI: 100	NR	Radionecrosis (30)
19	Pike et al., 2017 [45]	WBRT (36) SRS (73)	WBRT: 30 SRS: 20	Before: 78 After: 59	PEMBRO, NIVO or IPI: NR	2	NR
20	Skrepnik et al., 2017 [46]	SRS (100)	21	NR	IPI: 100	4	NR
21	Stokes et al., 2017 [47]	SRS (50.3), WBRT (49.7)		NR	Not specified	NR	NR
22	Yusuf et al., 2016 [48]	SRS (100); WBRT (5.6)	18	Concurrent or after: 39 Before: 61	Anti-PD-1: 72 IPI: 28	NR	Radionecrosis (3.4)
23	Liniker et al., 2016 [49]	WBRT (78) SRS (22)	WBRT: 30 SRS: NR	Concurrent: 52	NIVO: 20	NR	G ≥ 3–4 (WBRT): cognitive changes (5), Stevens–Johnson syndrome (5), nausea (5), rash (10)
24	Olson et al., 2016 [50]	SRS (100)	20	Before or Concurrent: 54 After: 46	IPI: 100	4	G3 CNS toxicities (11), radionecrosis (7)
25	Qin et al., 2016 [51]	SRS (100)	20	Before: 100	IPI: 100	>1	Dermatologic (27), gastrointestinal (18), fatigue (11), nausea (9), anorexia (5)
26	Theurich et al., 2016 [52]	WBRT (62); SRS (62)	30 (WBRT); 20 (SRS)	NR	IPI: 89 (11 received RT in other sites + SNC RT)	4	Overall G3-4 (0)
28	Gerber et al., 2015 [53]	WBRT (100)	30	Before: 23 Concurrent: 46 After: 53	IPI: 100	4	G3-4 Cognitive changes (8)
29	Kiess et al., 2015 [54]	SRS (100)	21	Before: 41 Concurrent: 33	PI: 100	NR	Radionecrosis (11)
30	Tazi et al., 2015 [55]	SRS (100)		Before or Concurrent: 100	IPI: 100	4	Overall G3-4 (10);
31	Mathew et al., 2013 [56]	SRS (100)	20	Before: 16	IPI: 100	4	Intracranial hemorrhage (28), radionecrosis (0)
32	Silk et al., 2013 [57]	WBRT (48.5) SRS (51.5)	NR	Before: 64 After: 36	IPI: 100	NR	NR
33	Knisely et al., 2012 [58]	SRS (100) SRS (100)	NR	26: SRS after IT 41: SRS after IT 59: SRS before IT	IPI: 100	NR	Radionecrosis (11)

**Table 3:** Risk of bias assessment.

Study. Years	Selection (5 Score)				Comparability (2 Score)	Outcome (2 Score)		Total Score
	Representative Sample	Sample Size	No Respondents	Ascertainment of the Exposure	Based on Design and Analysis	Assessment of Outcome	Statistical Test	
Galli et al., 2019 [24]	1	1	1	1	2	1	1	8
Chen et al., 2018 [28]	1	1	1	1	2	1	1	8
Diao et al., 2018 [29]	1	1	0	2	2	1	1	8
Hubbeling et al., 2018 [30]	1	1	0	0	2	1	0	5
Rahman et al., 2018 [31]	1	1	1	2	1	1	1	8
Schapira et al., 2018 [32]	1	1	0	1	2	1	0	6
Nardin et al., 2018 [33]	1	1	0	0	1	1	1	5
Acharya et al., 2017 [34]	1	1	0	0	2	1	1	6
Ahmed et al., 2017 [35]	1	1	0	0	2	1	0	5
An et al., 2017 [36]	1	1	1	0	1	1	0	5
Anderson et al., 2017 [37]	1	1	0	2	2	1	1	8
Choong et al., 2017 [38]	1	1	0	2	2	1	0	7
Fang et al., 2017 [39]	1	1	0	2	2	1	1	8
Gaudy-Marqueste et al., 2017 [40]	1	1	0	2	2	1	1	8
Kaidar-Person et al., 2017 [41]	1	1	0	0	2	1	1	6
Kotecha et al., 2017 [42]	1	1	0	1	1	1	1	6
Parakh et al., 2017 [43]	1	1	0	0	2	1	1	6
Patel et al., 2017 [44]	1	1	1	1	1	1	1	7
Pike et al., 2017 [45]	1	1	0	1	2	1	1	7
Skrepnik et al., 2017 [46]	1	1	0	0	2	1	1	6
Stokes et al., 2017 [47]	1	1	0	2	2	1	1	8
Yusuf et al., 2016 [48]	1	1	0	2	1	1	1	7
Liniker et al., 2016 [49]	1	1	1	1	2	1	1	8
Olson et al., 2016 [50]	1	1	1	1	2	1	1	8
Qin et al., 2016 [51]	1	1	0	2	2	1	1	8
Theurich et al., 2016 [52]	1	1	0	0	2	1	0	5
Gerber et al., 2015 [53]	1	1	1	2	1	1	1	8
Kiess et al., 2015 [54]	1	1	0	1	2	1	0	6
Tazi et al., 2015 [55]	1	1	0	0	1	1	1	5
Mathew et al., 2013 [56]	1	1	0	0	2	1	1	6
Silk et al., 2013 [57]	1	1	0	0	2	1	0	5
Knisely et al., 2012 [58]	1	1	1	0	1	1	0	5

## Data Availability

Not applicable.
